# Mood Prediction of Patients With Mood Disorders by Machine Learning Using Passive Digital Phenotypes Based on the Circadian Rhythm: Prospective Observational Cohort Study

**DOI:** 10.2196/11029

**Published:** 2019-04-17

**Authors:** Chul-Hyun Cho, Taek Lee, Min-Gwan Kim, Hoh Peter In, Leen Kim, Heon-Jeong Lee

**Affiliations:** 1 Korea University College of Medicine Department of Psychiatry Seoul Republic of Korea; 2 Sungshin University Department of Convergence Security Engineering Seoul Republic of Korea; 3 Korea University College of Informatics Department of Computer Science and Engineering Seoul Republic of Korea

**Keywords:** mood disorder, circadian rhythm, projections and predictions, machine learning, digital phenotype, wearable device

## Abstract

**Background:**

Virtually, all organisms on Earth have their own circadian rhythm, and humans are no exception. Circadian rhythms are associated with various human states, especially mood disorders, and disturbance of the circadian rhythm is known to be very closely related. Attempts have also been made to derive clinical implications associated with mood disorders using the vast amounts of digital log that is acquired by digital technologies develop and using computational analysis techniques.

**Objective:**

This study was conducted to evaluate the mood state or episode, activity, sleep, light exposure, and heart rate during a period of about 2 years by acquiring various digital log data through wearable devices and smartphone apps as well as conventional clinical assessments. We investigated a mood prediction algorithm developed with machine learning using passive data phenotypes based on circadian rhythms.

**Methods:**

We performed a prospective observational cohort study on 55 patients with mood disorders (major depressive disorder [MDD] and bipolar disorder type 1 [BD I] and 2 [BD II]) for 2 years. A smartphone app for self-recording daily mood scores and detecting light exposure (using the installed sensor) were provided. From daily worn activity trackers, digital log data of activity, sleep, and heart rate were collected. Passive digital phenotypes were processed into 130 features based on circadian rhythms, and a mood prediction algorithm was developed by random forest.

**Results:**

The mood state prediction accuracies for the next 3 days in all patients, MDD patients, BD I patients, and BD II patients were 65%, 65%, 64%, and 65% with 0.7, 0.69, 0.67, and 0.67 area under the curve (AUC) values, respectively. The accuracies of all patients for no episode (NE), depressive episode (DE), manic episode (ME), and hypomanic episode (HME) were 85.3%, 87%, 94%, and 91.2% with 0.87, 0.87, 0.958, and 0.912 AUC values, respectively. The prediction accuracy in BD II patients was distinctively balanced as high showing 82.6%, 74.4%, and 87.5% of accuracy (with generally good sensitivity and specificity) with 0.919, 0.868, and 0.949 AUC values for NE, DE, and HME, respectively.

**Conclusions:**

On the basis of the theoretical basis of chronobiology, this study proposed a good model for future research by developing a mood prediction algorithm using machine learning by processing and reclassifying digital log data. In addition to academic value, it is expected that this study will be of practical help to improve the prognosis of patients with mood disorders by making it possible to apply actual clinical application owing to the rapid expansion of digital technology.

## Introduction

### Background

Mood disorders, such as major depressive disorder (MDD) and bipolar disorder (BD), are common, with recurrent mood episodes and substantial chronicity. Patients with mood disorders suffer from a high disease burden, disrupted functional levels, and increased suicide risk [1,2]. It is crucial to use a coordinated approach to enhance prognosis by proactively managing symptoms and preventing recurrences. For successful prognosis enhancement, a new therapeutic approach is needed to assess, analyze, and manage the patient’s daily condition, in addition to conventional pharmacotherapeutic and psychotherapeutic approaches.

The circadian rhythm mechanism has been identified as an important factor in the onset and aggravation of mood disorders [3-5]. It has been reported that disturbances in circadian rhythms could be a unique clinical manifestation of mood disorders, and phase shift of circadian rhythms can serve as a marker for mood disorders [6,7]. Digital technology and machine learning have recently shown remarkable progress, bringing substantial changes into the lives of individuals [8]. In medicine, the use of digital phenotypes obtained with wearable technology or mobile devices has been reported [8-10]. Within psychiatry, research based on digital technology and machine learning has recently been introduced. This new research methodology is expected to overcome many limitations of existing psychiatric research through the precise analysis of clinical information obtained from various digital phenotypes. Digital phenotyping allows us to more closely and continuously measure information on a variety of biometrics, such as mood, activity, heart rate, and sleep, in the patient’s daily life and to connect these with clinical symptoms.

### Objectives

Using chronobiological concepts of mood disorders, in this prospective study, we collected long-term clinical mood logs and passively collected data on activity, sleep, light exposure, and heart rate in patients with mood disorders. Data were collected continuously through wearable devices and mobile technologies. We then analyzed the data to determine whether mood states or episodes could be predicted using only the automatically recorded data, without any knowledge on mood information, by machine learning.

## Methods

### Recruitment and Study Design

From March 2015 to December 2017, 55 patients (27 females and 28 males) diagnosed with a major mood disorder (MDD=18 subjects, BD I=18, and BD II=19) according to the Diagnostic and Statistical Manual of Mental Disorders, Fifth Edition (DSM-5), [11] were recruited from the Korea University Anam Hospital as part of the Mood Disorder Cohort Research Consortium (MDCRC) study (ClinicalTrials.gov: NCT03088657). Each patient had different days of participation period during the entire study period. The MDCRC study is a multicenter prospective observational cohort study investigating early-onset mood disorders in Korea, and its design and protocol have been reported previously [12]. The average age (SD) of the patients, age at first onset of mood disorder, and age at first psychiatric treatment was 25.92 (SD 4.78), 17.87 (SD 4.80), and 20.69 (SD 4.13) years, respectively (Multimedia Appendix 1). The study was approved by the Institutional Review Board of Korea University Anam Hospital and conducted in accordance with the Declaration of Helsinki. All participants provided informed written consent before enrollment after receiving a full explanation of the study.

### Assessment

In addition to standard clinical assessments conducted at regular intervals, including demographic and clinical data from investigators and patients, we provided an eMoodchart smartphone app developed on our own and a wearable activity tracker (Fitbit Charge HR or 2, Fitbit Inc). The smartphone app had an alert set for 9:00 o’clock every night, when patients recorded a simple, intuitive assessment of their daily mood state (−3 to +3) on the eMoodchart app. At every clinical assessment, a clinician reviewed the eMoodchart and determined the mood episodes that had occurred since the previous clinic visit. The between-visit mood episode evaluation was based on the eMoodchart and the patient interview according to DSM-5 [12]. For the comparison of basic features according to mood states, daily mood scores (−3 to +3) were converted to the absolute mood score (AMS; 0 to 3). When using the original recorded mood score as it is for analysis, it was difficult to reflect all kinds of mood states such as elated, depressed, or mixed, and it could add complexity to the overall trends. For the overall trend analysis, we decided to simply rearrange the mood to be stable or unstable in 2 directions. In other words, the higher the AMS, the mood can be regarded as worse and unstable (more depressed or more manic), and the lower the AMS, the mood can be regarded as more stable. For smartphones using the Android operating system (40 patients), the app could also detect light exposure, using a built-in sensor. The activity trackers, worn continuously, collected passive data related to activity, sleep, and heart rate, which were then obtained by the researchers from the Fitbit cloud server. The practicality and validity of using the Fitbit series for clinical research [13] and clinical results have been reported elsewhere [14]. As the smartphones are easily and frequently used in everyday life, there have been previous studies using the built-in sensors in smartphones [15-17].

### Datasets

During data collection, missing data were occasionally encountered for a variety of reasons (failure to complete the eMoodchart and removal of the activity tracker continuously over 24 hours). During the data collection period, we could originally collect 17,542 sample days from 55 patients, but the total number of 2003 days remained after removing the days with any single missing variable. In our analysis, we used only the complete dataset of 2003 days. Our dataset for prediction modeling has 130 variables (features) plus a class label of the mood state. We excluded the entire row if the row had a column with any missing value among the 131 columns. Among all the missing value counts, 16.8% was about light-related features, 9.1% was about step-related features, 43.9% was about sleep-related features, 29.6% was about heart rate–related features, and 0.3% was about the mood score record; perhaps, many people did not want to wear the Fitbit during sleep time. Heart rate–related features need the past 48-hour sequential data to be computed so that those features are not resistant against some missing data.

It has been reported that mood state can be affected by the disruption of circadian rhythms [6,7]; therefore, we focused on identifying a set of features that would capture such disruptions. To achieve this, we focused on basic features derived from the 4 main data collection categories: (1) light exposure, (2) steps, (3) sleep, and (4) heart rate (Multimedia Appendix 2).

To calculate light exposure, we took the average value of light exposure levels observed during 2 timeslots of interest: bedtime and daytime. As the lengths of day and night change according to the seasons, *bedtime* was defined as the period from 8 hours before sunrise until sunrise the next day and *daytime* was defined as the period from sunrise to sunset each day. It is not easy to adequately reflect seasonal changes, but because light exposure in the early morning is the most important time-giver (zeitgeber) in the daily circadian rhythm, sunrise time is a key criterion for defining *daytime* and *bedtime*. Therefore, according to the seasonal changes, *bedtime* was set at a constant total sleep time but variable at the time of sleep onset and wake up and *daytime* was set at total times when a person could be exposed to sunlight. To measure activity levels, we collected step data that was calculated as total steps within the bedtime and daytime timeslots. Sleep data such as sleep length and quality and sleep onset and offset were also obtained from the Fitbit report. With regard to heart rate, variations in heart rate follow a circadian rhythm, with elevated rates during the daytime and lower rates at night. A cosinor analysis (cosine curve fitting) was performed on 48 consecutive hours of heart rate data, and 4 representative parameters were generated: amplitude, acrophase (*peak*), mesor (*mean*), and r-squared value (*strength*).

Finally, we extracted extended features from the 4 basic categories, which integrated data across multiple days. In constructing the prediction model, these features are used as predictors for mood state or episode. To predict mood in the near future, it can be helpful to look at snapshots of previous days; perhaps, people could be affected by mood changes in these preceding days. Therefore, we extended the daily snapshot feature to simultaneously include the previous consecutive 3, 6, and 12 days. For example, if today’s date is d, then the mean value of the past 3 days would be from d-2 to d. In this way, the SD (*stdev*) and gradient coefficient (ie, a parameter gained from linear regression, *gradient*) can be computed for the extended features. The names of all features in the across-period perspective had a suffix including one of the 4 element names. The suffix terms also included the 3 elements describing the statistical perspective for the given period: *mean*, *stdev*, and *gradient*. Ultimately, we acquired 130 features (=13 basic features for every day+[13 basic features×3 types of the past periods×3 types of statistics for those periods]) for the data collected each day.

### Development and Verification of the Mood State and Episode Prediction Algorithms

To train the mood prediction model, we used a supervised learning algorithm, random forest [18], that operates by constructing a multitude of decision trees at training time and outputting a class that is the mode of the classes of the individual trees. The random forest algorithm requires a training dataset that consists of a feature vector set, *X=x*_*1*_*, …, x*_*n*_, with a corresponding class set, *Y=y*_*1*_*, …, y*_*n*_, where *n* is the number of training data samples (ie, n=2003 in our study). The feature vector *x*_*i*_ has the form f_1_, f_2_, …, f_m_, where m is 130 and f_i_ has a feature value of the circadian rhythm. The class variable *y*_*i*_ has one of 2 mood states: *biased mood state* or *neutral mood state* for mood state prediction within the next 3 days and has one of 4 episodes: *depressive episode (DE)*, *manic episode (ME)*, *hypomanic episode (HME)*, or *no episode (NE; same as euthymic period)* for mood episode prediction. The mood state was defined as *neutral mood state* if the average AMS for the following 3 days is within the bottom 50% (low AMS) of all the observed AMS. Conversely, the mood state was defined as *biased mood state* if the average AMS for the following 3 days is within the top 50% (high AMS). The mood episode was determined in the *between-visit mood episode evaluation* conducted by the clinician [12]. Patients in the study experienced 57 DEs (major: 46, minor: 3, and brief: 8), 11 MEs, and 13 HMEs.

Performance of the trained prediction model was evaluated by assessing the model’s accuracy, sensitivity, specificity, and the area under the curve (AUC) [19]. In a machine learning evaluation process, some portion of data is used for model training and the other portion is used for the model test. Training data should not include future measurements relative to the test data. To take into account such a temporal nature of the data and get a reliable evaluation statistic, we designed the model evaluation process as follows: first, data were sorted over the timeline. For an arbitrary time t on the timeline, a prediction model was trained using data on days d[t-p, t] and tested using data on days d[t+1, t+q], where p and q are the time period of days for model training and for model test, respectively. It is possible that the model performance can be changed depending on a different size of p or q. Therefore, to find a proper setting, we repeated and monitored experiments with changing p from 3 to 300 days and changing q from 3 to 30 days. Consequently, *P*=18 and q=3 were found as the best combination in our experiment setting (Multimedia Appendix 3), which implies that a short period such as 3 days is the most reasonable and effective setting in terms of predicting any distant future mood in our experiment. Therefore, for the performance evaluation of our proposed prediction model, we used the found parameter setting of p and q throughout the paper. Second, to get a reliable evaluation result, we needed to repeat enough evaluation rounds (ie, a round of model training and model test) so that we repeatedly measured performance metrics by moving t from the beginning to the end of the data over the timeline with the found parameter setting. Thus, the reported figures of sensitivity, specificity, accuracy, and AUC in the paper are average statistics from the repeated evaluation rounds. Finally, for a comparison between a general model and personalized model, a general model was developed using other people of the whole data and a personalized model was developed using only individual data. For data processing and model evaluation, we used a Python library tool, scikit-learn [20].

## Results

### Comparison of Main Basic Features According to Mood State

**Figure 1:**
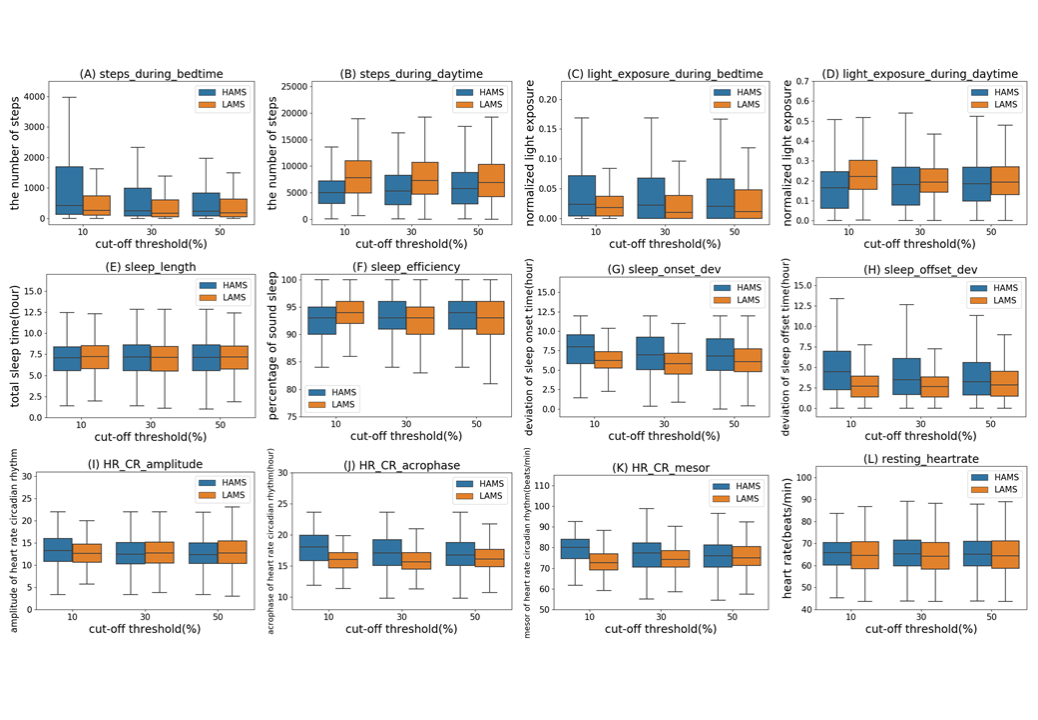
Comparison of basic feature distributions between high and low absolute mood score (HAMS and LAMS) groups. HAMS and LAMS were grouped into the upper or lower 10%, 30%, and 50% thresholds of the distribution. For each corresponding threshold group, we compared the main basic features of activity, light exposure, sleep, and heart rate (HR) related to the circadian rhythm (CR). The number in the parentheses on the horizontal axis means a cut-off threshold to distinguish HAMS and LAMS. (A) Steps_during_bedtime and (B) steps_during_daytime refer to activity levels of subjects during bedtime or daytime in each threshold HAMS or LAMS group. (C) Light_exposure_during_bedtime and (D) light_exposure_during_daytime refer to relative level of light exposure of subjects during bedtime or daytime. (E) Sleep_length and (F) sleep_efficiency refer to total sleep time (hours) and sleep efficiency (%), and (G) sleep_onset_dev and (H) sleep_offset_dev refer to regularity of sleep onset and offset time. (I) HR_CR_amplitude, (J) HR_CR_acrophase, and (K) HR_CR_mesor refer to the value of amplitude, acrophase, and mesor of cosine curve fitted HR, respectively. (L) resting_heart rate refers to the lowest HR at resting state during each day in the samples.

To confirm the appropriateness of the variables processed from a circadian rhythm perspective, we performed an exploratory review of the basic features by comparing them according to mood state. By sorting the collected data according to the AMS (0~3), mood state categories were created according to the AMS distribution and features belonging to them were compared. High and low AMS (HAMS or LAMS, respectively) days were grouped into the upper or lower 10%, 30%, and 50% thresholds of the distribution Therefore, the sum of the upper 50% of HAMS and the lower 50% of LAMS becomes the whole distribution of the AMS for each day of data. As presented in Figure 1, for each corresponding pair of threshold groups, we compared basic digital phenotypic features.

Activity and light exposure during bedtime showed a higher tendency in the HAMS groups than the LAMS groups. Conversely, activity and light exposure during daytime showed a higher tendency in LAMS (Figure 1). Interestingly, total sleep time and sleep quality did not show meaningful differences between the groups (Figure 1), although the regularity of sleep onset and offset times were disrupted in the HAMS groups (Figure 1), indicating that the regularity of the sleep-wake cycle is closely related to mood state. When the heart rate circadian rhythm was analyzed, it was found that the acrophase showed a remarkable difference between the HAMS and LAMS groups (Figure 1), suggesting that a misaligned or shifted heart rate acrophase could be a useful feature for determining mood state.

### Performance Evaluation of the Mood State Prediction Model (Neutral or Biased Mood State)

**Figure 2:**
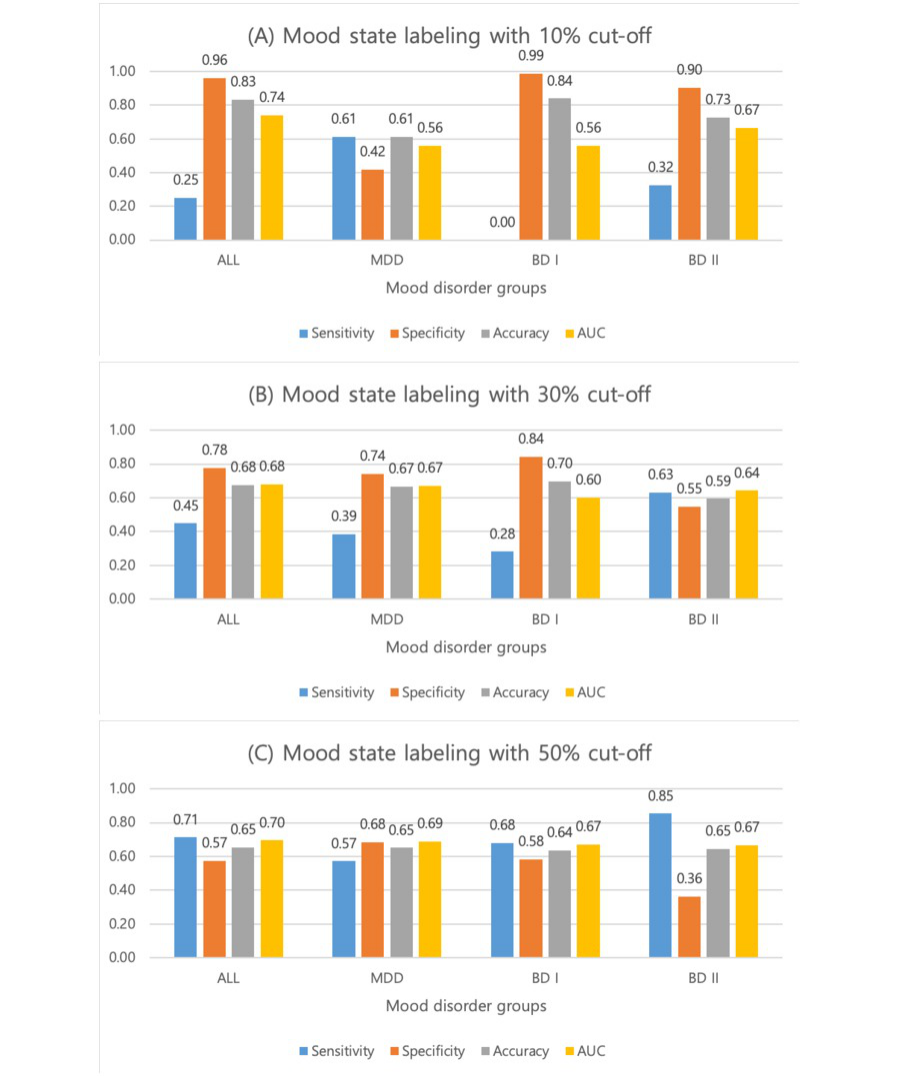
The performance evaluation of the mood state prediction model. The mood state prediction model outputs one of 2 mood states (ie, biased mood state or neutral mood state) and whether the model outcomes that were correctly matched with the ground truth (ie, the known actual mood states) was tested. The mood performance was evaluated in terms of the 4 performance evaluation metrics: sensitivity, specificity, accuracy, and area under the curve with the 3 different ground truth labeling criterion: 10%, 30%, and 50% cut-offs in absolute mood score distribution. (A) The performance evaluation result in the case of mood state labeling with 10% cut-off, (B) the performance evaluation result in the case of mood state labeling with 30% cut-off, and (C) the performance evaluation result in the case of mood state labeling with 50% cut-off. MDD: major depressive disorder; BD I: bipolar I disorder; BD II: bipolar II disorder; AUC: area under the curve.

In model construction, we used 2-class labels for distinguishing a mood state, which were determined by a 10%, 30%, and 50% cut-off threshold (eg, 10% of the highest AMS is labeled as *biased mood state* and the rest of the 90% AMS is labeled as *neutral mood state* in the 10% cut-off case). We tested the model performance in the 3 different conditions of thresholds. As seen in Figure 2, we confirmed that the mood state prediction model performed better than a random prediction model, as the AUC values for the 3 patient groups, as well as for all groups combined, were all higher than 0.5. In the case of mood state labeling with a 50% cut-off (Figure 2), the prediction accuracy for all patients and for patients with MDD, BD I, or BD II was 65%, 65%, 64%, and 65%, respectively. Sensitivity was 71%, 57%, 68%, and 85%, specificity was 57%, 68%, 58%, and 36%, and AUC values were 0.7, 0.69, 0.67, and 0.67 for predicting mood states in all patients and in MDD, BD I, and BD II patients, respectively. Note that the ROC curves of Figure 2 presented in Multimedia Appendix 4 and additional information about the variance of the model performance in each evaluation round are reported in Multimedia Appendix 5. The number of samples used in the model construction for each case is reported in Multimedia Appendix 6.

To understand the quality of predictions using partially observed covariates of features, we evaluated each performance of the partial model construction with the whole patient data, supposing that some missing features were removed. The AUC performance of each partially constructed model was 0.684 without steps-related features, 0.687 without sleep-related features, 0.683 without heart rate–related features, and 0.683 without light-related features (more details present in Multimedia Appendix 8). In the impact analysis of missing features, heart rate– and light-related features were of a highly negative impact in terms of performance reduction.

**Figure 3:**
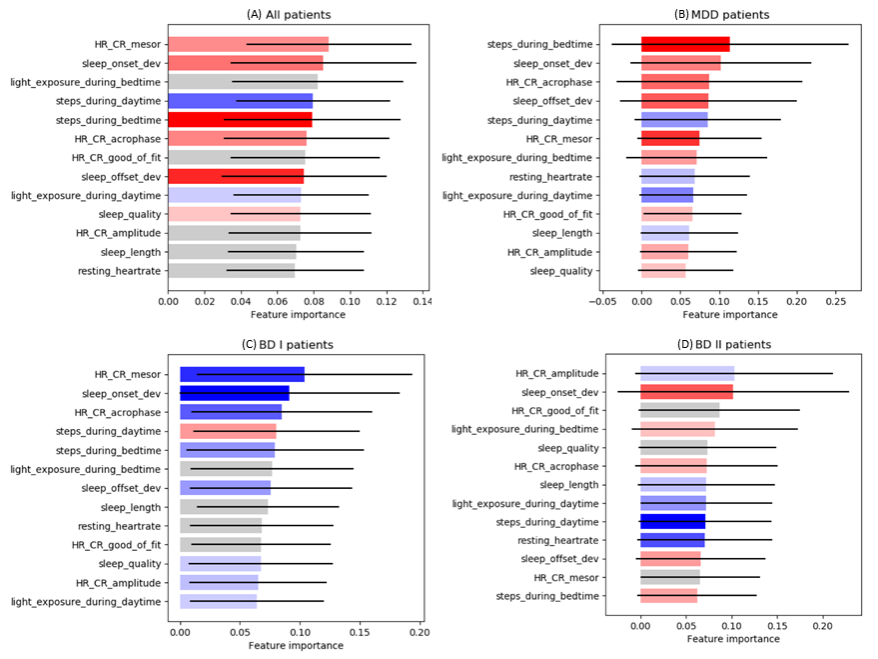
Representative influential features of the mood state prediction model. The mood state prediction model uses several categories of features and different features have different contribution to prediction performance. Each of the feature categories was compared in terms of its importance (contribution perspective). Each bar in the graph means relative importance of a feature category compared to the other bars. The black sold lines at the end of each bar means one standard deviation error range, and the color coding of each bar means the direction of feature effect; therefore, sum of all the bar lengths is one (100%). (A) The comparison analysis was conducted with data from all patients. (B) The analysis was conducted only with data from major depressive disorder patients. (C) The analysis was conducted only with data from bipolar I disorder patients. (D) The analysis was conducted only with data from bipolar II disorder patients. MDD: major depressive disorder; BD I: bipolar I disorder; BD II: bipolar II disorder; HR: heart rate; CR: circadian rhythm.

**Figure 4:**
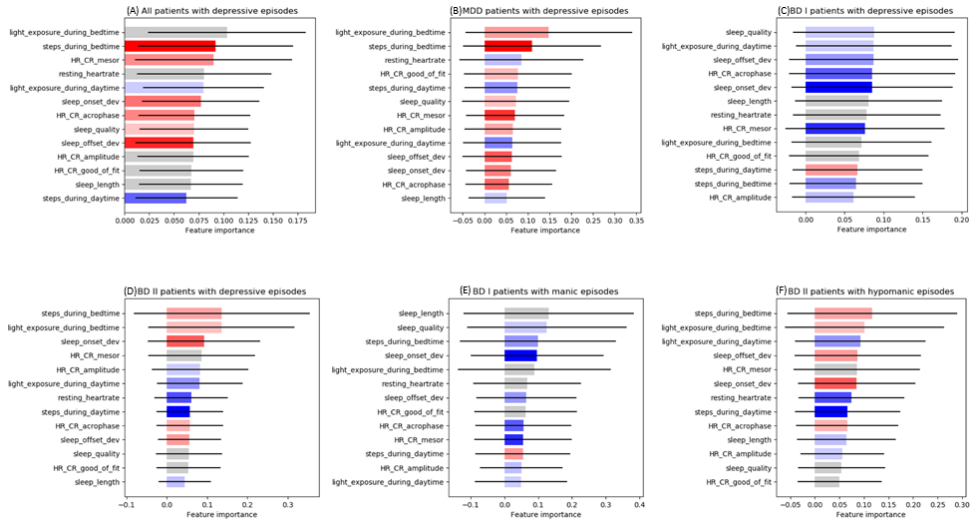
Representative influential features of the mood episode prediction model. The mood episode prediction model uses several categories of features, and different features have different contributions to prediction performance. Each of the feature categories was compared in terms of its importance (contribution perspective). Each bar in the graph means relative importance of a feature category compared with the other bars. The black sold lines at the end of each bar means 1 SD error range, and the color coding of each bar means the direction of feature effect; therefore, the sum of all the bar lengths is one (100%). (A) The comparison analysis was conducted with data from all patients with depressive episodes. (B) The analysis was conducted only with data from patients with major depressive disorder with depressive episodes. (C) The analysis was conducted only with data from patients with bipolar type I disorder with depressive episodes. (D) The analysis was conducted only with data from patients with bipolar type II disorder with depressive episodes. (E) The analysis was conducted only with data from patients with bipolar type I disorder with manic episodes. (F) The analysis was conducted only with data from patients with bipolar type II disorder with hypomanic episodes. MDD: major depressive disorder; BD I: bipolar type I disorder; BD II: bipolar type II disorder; HR: heart rate; CR: circadian rhythm.

To investigate the contribution of various features to the mood state predictions, we sorted the importance of influential features for prediction, depending on the patient group, as shown in Figure 3. The higher value in importance, the more frequently the feature is selected in a decision tree construction. To compute the feature importance, we used a Python library, scikit-learn [20], and referred to the code. In Figures 3 and 4, the color coding means the direction of feature effect was measured with Pearson correlation coefficients and the color-magnitude means relative strength of the correlation. Red color means a positive correlation with AMS (ie, the higher the feature value, the mood state tends to be more unstable.). Conversely, blue color means a negative correlation with AMS (ie, the higher the feature value, the less unstable the mood.). Gray color means the absolute coefficient values are less than 0.1, so it is hard to say any direction of the effect. Figures 3 and 4 have error variances of 1 SD with a solid black line at the end of each bar.

In the whole subject group and BD I group, the average circadian rhythm of heart rate (HR_CR_mesor) and deviation of sleep onset time (sleep_onset_dev) were the top influential features (Figure 3) and steps during bedtime were the most influential in the MDD group (Figure 3). Heart rate amplitude was the most influential in the BD II group (Figure 3).

### Performance Evaluation of the Mood Episode Prediction Model

As seen in Table 1, the mood episode prediction model was determined to perform better than a random prediction model according to AUC values. Basically, AUC cannot be calculated for multiclassification but for binary classification. Therefore, we merged all the samples not in the target class but into the remaining class for multiclasses of mood episodes. The average accuracies in all patients and in those with MDD, BD I, and BD II were 89.3%, 73.1%, 85.1%, and 78.5%, respectively. For all patients, prediction accuracy for NE, DE, ME, and HME was 85.3%, 87%, 94%, and 91.2%, respectively. Sensitivity was 93%, 48%, 25.2%, and 30.6%, specificity was 59.9%, 95.6%, 99.6%, and 99.6%, and the AUC value was 0.87, 0.87, 0.958, and 0.912 for predicting NE, DE, ME, and HME, respectively. For patients with MDD, the prediction accuracy was 75.1% and 71.2%, sensitivity was 93.5% and 40.9%, specificity was 39.5% and 87.8%, and the AUC value was 0.781 and 0.798 for NE and DE, respectively. For patients with BD I, prediction accuracy was 84%, 83.1%, and 88.3%, sensitivity was 95.4%, 24.6%, and 20.7%, specificity was 39.3%, 97%, and 99.2%, and the AUC value was 0.84, 0.839, and 0.933 for NE, DE, and ME, respectively. For patients with BD II, prediction accuracy was 82.6%, 74.4%, and 87.5%, sensitivity was 84.5%, 64.1%, and 66.9%, specificity was 76.2%, 72.2%, and 98.4%, and the AUC value was 0.919, 0.868, and 0.949 for NE, DE, and HME, respectively. The number of samples used in the model construction for each case is reported in Multimedia Appendix 6. Figure 4 provides information on the important influential features in prediction performance capability in the diagnostic groups.

**Table 1:** The performance evaluation of the mood episode prediction model.

Episodes and measures		All patients	Major depressive disorder	Bipolar type I disorder	Bipolar type II disorder
**No episodes (euthymic period)**					
	Samples, %	82	92.5	85	66.9
	Sensitivity	0.93	0.965	0.954	0.845
	Specificity	0.599	0.395	0.393	0.762
	Accuracy	0.853	0.751	0.84	0.826
	AUC^a^	0.87	0.781	0.84	0.919
**Depressive episodes**					
	Samples, %	12.3	7.4	11.8	18.2
	Sensitivity	0.48	0.409	0.246	0.641
	Specificity	0.956	0.878	0.97	0.722
	Accuracy	0.87	0.712	0.831	0.744
	AUC	0.87	0.798	0.839	0.868
**Manic episodes**					
	Samples, %	1.1	—^b^	3	—
	Sensitivity	0.252	—	0.207	—
	Specificity	0.996	—	0.992	—
	Accuracy	0.94	—	0.883	—
	AUC	0.958	—	0.933	—
**Hypomanic episodes**					
	Samples, %	4.4	—	—	14.8
	Sensitivity	0.306	—	—	0.669
	Specificity	0.996	—	—	0.984
	Accuracy	0.912	—	—	0.875
	AUC	0.912	—	—	0.949
Average accuracy		0.893	0.731	0.851	0.785

^a^AUC: area under the curve.

^b^Not applicable.

### General Model Versus Personalized Model

When constructing a machine learning model, the model is supposed to feed as much data as possible for the purpose of general knowledge learning. If a prediction model is learned by much data of the other people, it is called a general model in this paper. Conversely, if a prediction model is learned by personal data, it is called a personalized model. A general model can have comprehensive knowledge, but it is not specialized to an individual. A personalized model has specific knowledge especially for an individual. It is specialized to one so it is not applicable to the other one. It is a possible idea that a personalized model can improve the prediction accuracy of personal mood change. Therefore, as mood and related features showed various manifestations for each subject, we compared the performance of the personalized and general models in terms of accuracy for 4 prediction scenarios: (1) mood state for the next 3 days (*future mood state*), (2) DE, (3) ME, and (4) HME (Multimedia Appendix 7). For the future mood state, the personalized model outperformed the general model in 100% of the comparisons. The average degree to which the personalized model outperformed was 23.8%, meaning that all the cases of the personalized model predictions were 23.8% more accurate, on average, than those of the general model. In the scenario cases of episode prediction, the personalized models almost perfectly outperformed the general model as well.

## Discussion

### Principal Findings

In an exploratory review, we found several basic features from the passively collected data that showed a clear difference between the HAMS and LAMS groups. Light exposure is important because light is a central modulator of sleep, mood, and circadian rhythms [21]. Activity at appropriate times of the day and appropriate amounts could be one kind of social zeitgeber (*time-giver*) for the maintenance of circadian rhythms and mood [22]. Activity and light exposure, which are basic and important in terms of human circadian rhythms, consistently show clear and distinct differences according to the mood state. This suggests that it would be useful for patients with mood disorders to manage their activity levels and exposure to light to coordinate with their circadian rhythm to maintain a stable mood state. It is clinically significant that irregularities in the sleep-wake cycle are common in abnormal mood states [23]. The group differences in the heart rate acrophase identified in this study can be considered in the same context. Confirming the findings in Figure 1, we demonstrated that basic features related to circadian rhythms can meaningfully reflect the mood state.

The overall prediction accuracy for the mood state was relatively good. Interestingly, the sensitivity in BD II was markedly higher and the specificity lower than in other groups. This result may reflect characteristic features of BD II such as a close relationship to circadian rhythm disturbances as well as very common and sensitive mood changes in BD II. In the mood episode prediction model, overall prediction accuracy was quite good across all patient groups. The sensitivity to predict a DE was low across all groups except BD II (ie, all patients, MDD patients, and BD I patients). This may be because a DE is likely to be influenced by diverse factors including disturbance of circadian rhythms, socioeconomic stress, and interpersonal problems [6,24]. The prediction performance was markedly better in the BD II group for all episodes. In other words, the HME and DE in patients with BD II showed clearly distinguishable features compared with the euthymic period. BD II has been proposed as a distinctive major mood disorder from BD I or MDD, in terms of brain abnormalities, a number of previous mood episodes, seasonal aggravations, the circadian rhythm, depressive admixtures, and comorbidity [6,25-28]. The results of this study also show that BD II exhibits more discriminating characteristics than other major mood disorders. In particular, the superiority of the mood prediction algorithm based on the circadian rhythm suggests indirectly that BD II is likely to be affected by the disturbance of the circadian rhythm compared with mood episodes of other mood disorders. On the contrary, it is also possible that better predictions for BD II might be an artifact of the relatively well-balanced dataset for BP II and the fact that standard splits in random forests are not well-suited for the imbalanced classification. We will need further research for this possibility in the future.

Circadian disturbances have been reported in MDD including diurnal mood variation [29], core body temperature abnormality [30], changes in secretion of melatonin and cortisol [31], circadian rhythm alteration induced by antidepressants [32], and sleep-wake cycle disruption [31,33]. BD has even more robust results reported than MDD in relation to circadian rhythms, from an association with circadian gene variants [34], through sleep and circadian phenotypes [4,6,7], to the therapeutic approaches focusing on circadian rhythms and sleep [35]. Previous researchers have reported that seasonal variations in mood, behavior, and diurnal preference, and irregular bed-rise times, are closely related to BD, suggesting the importance of circadian rhythms in BD [36]. In particular, studies showed a closer association with seasonality in BD II than in BD I [37] and a greater chronotherapeutic effect in BD II [38].

Although the diagnosis of a mood disorder may be the same, clinical features vary from person to person. Automatically and passively recorded data from a diverse range of routine lives can directly or indirectly provide rich information reflecting each person’s psychiatric characteristics. Clinical symptoms are assessed primarily through interviews or psychiatric scales, which are dependent on reports from the patient or caregiver and are prone to recall errors and subjective bias. Continuously collected digital log data can provide a personalized upgrade to traditional clinical information. The mood prediction algorithm from this study provides a timely opportunity for the practical application of these data to treatment, especially for preventing acute mood episodes and managing daily mood states. The rapid development of information and communication technology (ICT) will present new therapeutic paradigm shifts for both clinicians and patients and help to fill the care gap in existing conventional treatments.

Mood follows a flow, so if patients record their own mood state every day, it can be a powerful predictor of future mood. However, recording daily mood requires ongoing attention and effort. As adherence is a key issue in mood disorder treatment, compliance would be improved if a patient’s condition could be managed and analyzed without any special effort or action. Using a smartphone and wearable device is a simple and convenient way to collect data to predict the mood state or pathological mood episodes.

Many studies have been conducted so far to predict mood or stress with data collection from smartphones such as the number of phone calls and text messages communicating with other people [16], entropy of subject’s location changes based on the global positioning system (GPS) [39], behavioral movement detection from accelerometer sensors [17,40], ambient light and noise sounds [17], and the paralinguistic feature of speech from smartphones [40]. LiKamWa et al used smartphone sensors to predict mood change in their study for 32 subjects and 2 months [16]. They analyzed the number and length of calls, short message service (SMS) text messages, and email communications; the usage number and pattern of apps; history of Web browser connections; and change of location information, reporting a prediction accuracy with 66%. After using the personalized prediction model, they could improve the accuracy up to 93%. Ma et al analyzed location information, user action and movement detection, ambient light and sounds, predicting the mood state with 50% accuracy in their study with 15 subjects and for 30 days [17]. One of the well-known projects for mood prediction study using smartphone-based sensors is the MONARCA project [41]. In the project, 12 actual BD were studied for 12 weeks. The mood prediction accuracy was achieved at 72% to 81% by using an accelerometer sensor and GPS-based location information. The prediction accuracy could be improved some more by including features of phone speech analysis. Gravenhorst et al also found from an extra study that higher use of social and entertainment apps was associated with lower stress and irritability [41]. Palmius et al could distinguish the mood depressive state from the nondepressive state with 85% accuracy by using features of GPS information including the entropy and circadian rhythm [42]. Carr et al studied if variability in phase and amplitude of the diurnal rhythm is related to variation of mood in bipolar and borderline personality disorder [43]. They investigated mood and diurnal variation for 4 days in 20 outpatients with BD, 14 with borderline personality disorder, and 20 healthy controls using a smartphone app, portable electrocardiogram, and actigraphy, reporting that for borderline personality disorder, there was a pattern of positive correlations between mood variability and variation in activity, sleep, and heart rate.

The previous studies were rather based on smartphone built-in sensors than on wearable devices. Smartphone sensors would be useful, but they have some limitations. First, smartphone is portable but not wearable. Therefore, even though smartphone is very easily applicable, it is not directly attached to the body so it is hard to collect data continuously without missing points over the timeline. Second, a privacy issue is serious. Many studies are depending on collecting data such as phone calls, SMS text messages, and GPS information, which are very sensitive and hard to be collected for a long time. However, a psychiatric study of mood prediction usually should need a long-time follow-up. The existing related studies have reported quite promising results in terms of mood prediction. However, they are mostly not analyzing actual BD patients but studying students or ordinary people without a mental disorder under the laboratory experiment setting or under an artificially instrumented environment. The number of analyzed people is limited to a small size and the length of study is not more than an annual period.

In contrast, our study was based on big data collection and analysis for about 2 years from 55 actual major mood disorder patients, which is reliably measured by a wearable device (a popular commercial product). To our knowledge, the proposed rhythm features (Multimedia Appendix 2) are unique and have never been tried before in model construction of the existing mood prediction studies. The accuracy of the proposed model performance is also reasonable compared with the existing related studies.

This study has several clinical strengths. First, we prospectively collected a vast amount of data for about 2 years from study subjects with mood disorders, accumulating 52,884 days of samples. Second, automatically passively recorded digital data from patients were collected using ICT, and the collected data were categorized, processed, and analyzed according to our hypotheses, regarding the influence of circadian rhythms, to obtain 130 daily features. As a result, from 260,390 to 6,874,920 total features (in the case of a complete dataset=2003 sample days×130 features and an incomplete dataset=52,884 sample days×30 features) were available for machine learning training and played an important role in improving predictability. Third, clinically significant prediction performance for a mood state or pathological mood episode was demonstrated using automatically recorded passive digital log data, in the absence of clinically derived mood information. Finally, the personalized prediction algorithm showed the potential to apply precision medicine principles to psychiatry.

### Limitations

This study has some limitations. First, more intrinsic and preemptive genetic and biological assessments related to circadian rhythms were not included, as this would have required a more complex study design and analysis methods. We are planning to include these in future research. Second, the prediction capabilities were not high in some cases. However, the purpose of this study was not to diagnose mood disorders but to predict the mood state or episodes in patients with a diagnosed mood disorder, to improve the prognosis of patients with mood disorders by self-monitoring and self-care of moods and circadian rhythms in daily life through the mood prediction system developed in this study. Third, when analyzing the mood state, HAMS and LAMS were applied separately according to the absolute score of mood. This might not be an accurate reflection of the mood state, as we did not apply the high and low mood scores correctly. However, it was not easy to verify the accuracy of daily mood scores unlike the mood episodes, because the subjective mood score recorded by an individual may vary according to individual characteristics and tendencies. Finally, we reported the model performance results for individuals from our collected dataset only. Therefore, it is not sure how well the results will generalize to a new population. One way of addressing this properly would be by using cross-validation for different unseen patients. Some individuals would be selected for testing and others for training. The model would be trained using the training individuals and evaluated using the test individuals, and the procedure would be repeated for multiple splits as in the usual cross-validation. Thus, it is necessary for an additional future study to test and secure more external validity.

### Conclusions

To our knowledge, this is the first study to develop a prediction system using only passive digital phenotypes from patients with mood disorders for a prolonged period of time. We have developed and verified mood state and pathological mood episode prediction algorithms using only automatically recorded passive data. On the basis of the results of this study, mood prediction algorithms can be applied therapeutically to improve clinical outcomes and the prognosis of patients with mood disorders. This study is just the first step toward future digital and precision medicine in the psychiatric field [44]. In future, a revolutionary change in psychiatric treatment will occur through the establishment of an integrated platform with genetic information and biological therapy.

## Appendix

Multimedia Appendix 1 Demographic and clinical information of the subjects.

Multimedia Appendix 2 The proposed basic features to check circadian rhythm from the automatically measured passive digital log data of patients with mood disorders.

Multimedia Appendix 3 Different parameter setting affects model performance. The legend means the model test period q and the horizontal axis means the model training period p has 1 time of q days, 2 times of q days, 3 times of q days, and so on. The vertical axis means the area under the curve result after evaluating model performance with a parameter combination of p and q.

Multimedia Appendix 4 The entire receiver operating characteristic curves of the main manuscript are presented here for clearer understanding of some unreported calibration in sensitivity and specificity.

Multimedia Appendix 5 This is a Kernel Density Estimate plot describing variance of the model performance that was reported in the main manuscript (Mood state labeling with 50% cut-off) for the mood disorder group ALL. The horizontal axis presents the area under the curve (AUC) distribution and the vertical axis presents density of each AUC observation from multiple performance evaluation rounds. The total number of evaluation rounds is 668.

Multimedia Appendix 6 The number of samples used in the mood state and mood episode prediction.

Multimedia Appendix 7 Prediction performance comparison between personalized model versus general model.

Multimedia Appendix 8 The area under the curve performance of each partially constructed model. The first column means a group of features that are missed in the model construction, and the second column is the number of features that are used in the model construction except the group of missing features. The rest of columns are the constructed model performance without using the missing features.

## Abbreviations

AMS: absolute mood score
AUC: area under the curve
BD: bipolar disorder
BD I: bipolar disorder type 1
BD II: bipolar disorder type 2
DE: depressive episode
DSM: Diagnostic and Statistical Manual of Mental Disorders
GPS: global positioning system
HAMS: high absolute mood score
HME: hypomanic episode
ICT: information and communication technology
LAMS: low absolute mood score
MDCRC: Mood Disorder Cohort Research Consortium
MDD: major depressive disorder
ME: manic episode
NE: no episode
