# Addendum to the Acknowledgements: Mood Prediction of Patients With Mood Disorders by Machine Learning Using Passive Digital Phenotypes Based on the Circadian Rhythm: Prospective Observational Cohort Study

**DOI:** 10.2196/15966

**Published:** 2019-10-03

**Authors:** Chul-Hyun Cho, Taek Lee, Min-Gwan Kim, Hoh Peter In, Leen Kim, Heon-Jeong Lee

**Affiliations:** 1 Korea University College of Medicine Department of Psychiatry Seoul Republic of Korea; 2 Sungshin University Department of Convergence Security Engineering Seoul Republic of Korea; 3 Korea University College of Informatics Department of Computer Science and Engineering Seoul Republic of Korea

**Keywords:** mood disorder, circadian rhythm, prediction, machine learning, digital phenotype, wearable device

The authors of “Mood Prediction of Patients With Mood Disorders by Machine Learning Using Passive Digital Phenotypes Based on the Circadian Rhythm: Prospective Observational Cohort Study” (J Med Internet Res 2019;21(4):e11029) missed an important source of funding in the Acknowledgments section — the Korea Health 21 R&D Project funded by the National Research Foundation of Korea (2017M3A9F1031220).

At the time of publication, the Acknowlegments section read as follows:

This independent research study was supported by the Korea Health 21 R&D Project funded by the Ministry of Health & Welfare, Republic of Korea (HM14C2606 and HI14C3212) and the National Research Foundation of Korea (2016M3C7A1904345). Most of all, the authors express their gratitude to the participants who took part in the study.

The revised Acknowledgments section now appears as follows:

This independent research study was supported by the Korea Health 21 R&D Project funded by the Ministry of Health & Welfare, Republic of Korea (HM14C2606 and HI14C3212), and the National Research Foundation of Korea (2016M3C7A1904345 and 2017M3A9F1031220). Most of all, we express our gratitude to the participants who took part in the study.

The correction will appear in the online version of the paper on the JMIR website on October 3, 2019, together with the publication of this correction notice. Because this was made after submission to PubMed, PubMed Central, and other full-text repositories, the corrected article has also been resubmitted to those repositories.

