# Supplementary material for: Mood Prediction of Patients With Mood Disorders by Machine Learning Using Passive Digital Phenotypes Based on the Circadian Rhythm: Prospective Observational Cohort Study
Source: J Med Internet Res. 2019 Apr 17;21(4):e11029. doi: 10.2196/11029 (PMC6492069; doi:10.2196/11029)
Supplement: Multimedia Appendix 1 [file jmir_v21i4e11029_app1.docx]

**Supplementary Table 1.** Demographic and clinical information of the subjects.

| Subject No. | Diagnosis | Gender | Age | Height (cm) | Weight (kg) | Education level | Marital status | No. of major depressive episodes | No. of manic episodes | No. of hypomanic episodes | Age of first onset | Age of first psychiatric treatment | No. of previous psychiatric administration | Age of first psychiatric administration |
| --- | --- | --- | --- | --- | --- | --- | --- | --- | --- | --- | --- | --- | --- | --- |
| 1 | Bipolar disorder, type Ⅱ | male | 29 | 175.6 | 75.7 | 16 | Unmarried | 15 | - | 1 | 13 | 13 | - | - |
| 2 | Bipolar disorder, type Ⅱ | female | 34 | 165 | 84.4 | 17 | Unmarried | 3 | - | 5 | 28 | 29 | - | - |
| 3 | Bipolar disorder, type Ⅰ | male | 39 | 177.6 | 80.3 | 16 | Unmarried | 20 | 3 | 10 | 10 | 30 | 3 | 30 |
| 4 | Bipolar disorder, type Ⅰ | female | 39 | 155.7 | 69 | 16 | Married | 20 | 6 | 30 | 15 | 18 | 2 | 18 |
| 5 | Bipolar disorder, type Ⅰ | female | 44 | 146.1 | 49.9 | 13 | Married | 10 | 4 | - | 17 | 17 | 4 | 31 |
| 6 | Bipolar disorder, type Ⅰ | male | 25 | 186.3 | 75.3 | 15 | Unmarried | - | 1 | - | 21 | 21 | 1 | 21 |
| 7 | Bipolar disorder, type Ⅰ | male | 27 | 166.2 | 59.0 | 16 | Unmarried | 3 | 3 | 5 | 20 | 20 | 3 | 20 |
| 8 | Bipolar disorder, type Ⅰ | female | 26 | 162.2 | 62.0 | 16 | Unmarried | 5 | 2 | 2 | 18 | 18 | 2 | 20 |
| 9 | Major depressive disorder | female | 21 | 149.0 | 67.1 | 12 | Unmarried | 3 | - | - | 15 | 16 | - | - |
| 10 | Major depressive disorder | male | 29 | 179.6 | 87.0 | 12 | Unmarried | 2 | - | - | 25 | 26 | - | - |
| 11 | Major depressive disorder | female | 28 | 161.0 | 56.2 | 16 | Unmarried | 5 | - | - | 20 | 21 | - | - |
| 12 | Bipolar disorder, type Ⅰ | female | 29 | 175.0 | 87.1 | 16 | Unmarried | 2 | 2 | - | 24 | 25 | 1 | 25 |
| 13 | Bipolar disorder, type Ⅱ | male | 25 | 170.0 | 71.6 | 14 | Unmarried | 20 | - | 10 | 14 | 17 | 1 | 18 |
| 14 | Bipolar disorder, type Ⅱ | female | 25 | 165.0 | 64.9 | 13 | Unmarried | 7 | - | 1 | 16 | 16 | 1 | 16 |
| 15 | Bipolar disorder, type Ⅱ | female | 26 | 162.0 | 60.7 | 14 | Unmarried | 1 | - | 2 | 20 | 22 | 1 | 23 |
| 16 | Bipolar disorder, type Ⅰ | female | 30 | 158.5 | 49.8 | 18 | Unmarried | - | 1 | - | 27 | 27 | 1 | 27 |
| 17 | Bipolar disorder, type Ⅰ | male | 26 | 166.9 | 87.3 | 15 | Unmarried | 3 | 2 | - | 21 | 23 | 1 | 23 |
| 18 | Bipolar disorder, type Ⅰ | male | 32 | 176.3 | 74.2 | 16 | Unmarried | 1 | 1 | - | 29 | 29 | 1 | 29 |
| 19 | Bipolar disorder, type Ⅰ | female | 21 | 160.6 | 59.9 | 13 | Unmarried | 15 | 3 | - | 14 | 16 | 5 | 16 |
| 20 | Bipolar disorder, type Ⅰ | female | 25 | 167.9 | 55.2 | 16 | Unmarried | 3 | 2 | - | 17 | 17 | 3 | 17 |
| 21 | Bipolar disorder, type Ⅰ | male | 24 | 176.7 | 72.7 | 13 | Unmarried | 8 | 5 | 13 | 12 | 16 | 5 | 16 |
| 22 | Bipolar disorder, type Ⅱ | male | 21 | 170.6 | 44.9 | 13 | Unmarried | 3 | - | 1 | 14 | 16 | 1 | 19 |
| 23 | Bipolar disorder, type Ⅱ | female | 23 | 162.4 | 58.1 | 14 | Unmarried | 15 | - | 7 | 17 | 20 | - | - |
| 24 | Major depressive disorder | male | 29 | 178.5 | 88.0 | 16 | Unmarried | 2 | - | - | 24 | 26 | - | - |
| 25 | Major depressive disorder | male | 23 | 181.3 | 114.6 | 13 | Unmarried | 1 | - | - | 19 | 19 | - | - |
| 26 | Bipolar disorder, type Ⅱ | female | 21 | 159.0 | 42.4 | 13 | Unmarried | 3 | - | 2 | 17 | 18 | - | - |
| 27 | Major depressive disorder | male | 24 | 176.4 | 51.0 | 15 | Unmarried | 7 | - | - | 18 | 19 | - | - |
| 28 | Bipolar disorder, type Ⅱ | male | 23 | 182.5 | 112.2 | 13 | Unmarried | 3 | - | - | 20 | 21 | - | - |
| 29 | Bipolar disorder, type Ⅰ | male | 27 | 185.3 | 84.2 | 15 | Unmarried | 3 | 2 | 8 | 15 | 17 | 2 | 17 |
| 30 | Bipolar disorder, type Ⅱ | female | 24 | 164.2 | 56.3 | 16 | Unmarried | 25 | - | 3 | 15 | 22 | - | - |
| 31 | Bipolar disorder, type Ⅱ | male | 26 | 176.5 | 114.9 | 14 | Unmarried | 1 | - | 2 | 15 | 22 | - | - |
| 32 | Major depressive disorder | male | 21 | 188.4 | 82.6 | 13 | Unmarried | 1 | - | - | 19 | 19 | - | - |
| 33 | Major depressive disorder | male | 26 | 174.2 | 76.2 | 16 | Unmarried | 2 | - | - | 24 | 24 | 2 | 24 |
| 34 | Major depressive disorder | male | 23 | 170.0 | 60.8 | 14 | Unmarried | 20 | - | - | 9 | 19 | 1 | 19 |
| 35 | Bipolar disorder, type Ⅱ | female | 26 | 163.4 | 52.7 | 17 | Unmarried | 10 | - | 4 | 21 | 24 | - | - |
| 36 | Bipolar disorder, type Ⅰ | male | 25 | 181.9 | 67.0 | 14 | Unmarried | - | - | 12 | 17 | 17 | - | - |
| 37 | Major depressive disorder | male | 28 | 184.5 | 74.7 | 18 | Unmarried | 4 | - | - | 25 | 25 | - | - |
| 38 | Major depressive disorder | male | 22 | 174.3 | 59.2 | 13 | Unmarried | 3 | - | - | 17 | 20 | - | - |
| 39 | Major depressive disorder | male | 29 | 168.3 | 66.8 | 20 | Unmarried | 1 | - | - | 26 | 26 | - | - |
| 40 | Bipolar disorder, type Ⅱ | female | 30 | 165.6 | 56.7 | 16 | Unmarried | 1 | - | 2 | 26 | 26 | - | - |
| 41 | Bipolar disorder, type Ⅱ | male | 23 | 182.0 | 52.0 | 15 | Unmarried | 3 | - | 3 | 13 | 22 | - | - |
| 42 | Major depressive disorder | male | 22 | 169.7 | 53.4 | 13 | Unmarried | 5 | - | - | 13 | 13 | - | - |
| 43 | Major depressive disorder | female | 26 | 165.7 | 73.0 | 16 | Unmarried | 7 | - | - | 20 | 23 | - | - |
| 44 | Bipolar disorder, type Ⅱ | female | 20 | 159.5 | 50.1 | 14 | Unmarried | 3 | - | 1 | 16 | 19 | - | - |
| 45 | Major depressive disorder | female | 23 | 163.8 | 66.9 | 16 | Unmarried | 3 | - | - | 17 | 21 | - | - |
| 46 | Bipolar disorder, type Ⅰ | female | 19 | 153.7 | 52.5 | 12 | Unmarried | 5 | 3 | 10 | 12 | 13 | 2 | 13 |
| 47 | Major depressive disorder | female | 25 | 160.0 | 52.4 | 16 | Unmarried | 4 | - | - | 17 | 17 | 1 | 17 |
| 48 | Bipolar disorder, type Ⅱ | male | 25 | 165.6 | 78.5 | 13 | Unmarried | 30 | - | 25 | 16 | 24 | - | - |
| 49 | Bipolar disorder, type Ⅰ | female | 21 | 160.9 | 49.0 | 14 | Unmarried | 5 | 2 | 3 | 11 | 20 | 2 | 20 |
| 50 | Bipolar disorder, type Ⅰ | female | 25 | 158.6 | 68.8 | 15 | Unmarried | - | 1 | - | 21 | 21 | 1 | 21 |
| 51 | Bipolar disorder, type Ⅱ | female | 26 | 162.0 | 54.0 | 16 | Unmarried | 5 | - | 5 | 14 | 21 | - | - |
| 52 | Bipolar disorder, type Ⅱ | male | 26 | 168.7 | 67.4 | 16 | Unmarried | 8 | - | 20 | 14 | 24 | - | - |
| 53 | Bipolar disorder, type Ⅱ | male | 27 | 167.0 | 90.9 | 15 | Unmarried | 6 | - | 20 | 20 | 26 | - | - |
| 54 | Major depressive disorder | female | 23 | 169.3 | 51.6 | 16 | Unmarried | 15 | - | - | 13 | 18 | - | - |
| 55 | Major depressive disorder | female | 20 | 156.4 | 60.6 | 14 | Unmarried | 5 | - | - | 12 | 19 | - | - |
