# Supplementary material for: Mood Prediction of Patients With Mood Disorders by Machine Learning Using Passive Digital Phenotypes Based on the Circadian Rhythm: Prospective Observational Cohort Study
Source: J Med Internet Res. 2019 Apr 17;21(4):e11029. doi: 10.2196/11029 (PMC6492069; doi:10.2196/11029)
Supplement: Multimedia Appendix 2 [file jmir_v21i4e11029_app2.docx]

**Supplementary table 2. The proposed basic features to check circadian rhythm (CR) from the automatically measured passive digital log data of patients with mood disorders.**

| **Category** | **Feature name** | **Description** | **Rationale** |
| --- | --- | --- | --- |
| Light exposure | light_exposure_during_bedtime | The cumulative amount of light exposure during the bedtime that means period from 8 hours earlier before sunrise to the time of sunrise. | Light is the most important trigger that synchronizes circadian rhythm of all the creatures on the earth. Being exposed to light at night is not desirable but that is quite recommended during daytime. |
|  | light_exposure_during_daytime | The cumulative amount of light exposure during the daytime that means period from the time of sunrise to sunset. |  |
| Step | steps_during_bedtime | The cumulative counts of steps during the bedtime | It is recommended to walk enough in the daytime and not to go around during bedtime to keep a sound circadian (sleep-wake) rhythm. |
|  | steps_during_daytime | The cumulative counts of steps during the daytime |  |
| Sleep | sleep_length | The time length of sleep | Sufficient and regular sleep is the important factors in achieving a sound circadian rhythm and relieving fatigue and stress. |
|  | sleep_quality | The quality score of sleep between 0 to 100. It is computed with (sleep length - restless sleep length) / sleep length |  |
|  | sleep_onset_dev | Deviation of sleep onset times is measured to check whether the sleep onset times are regular. The deviation means observed time distances from the moment, 8 hours ago before the sunrise time of the next day. |  |
|  | sleep_offset_dev | Deviation of sleep offset (wake-up) times is measured to check whether the sleep offset times are regular. The deviation means observed time distances from the sunrise time. |  |
| Heart rate (HR) | HR_CR_amplitude | The daily HR rhythm of cosine curve has its amplitude. Large amplitude means a clear rhythm curve with low HR in sleep and high HR in activity. | The HR contains important rhythmic information. HR falls when sleeping and goes up when activity increases. Therefore, it is ideal to have a good S-shaped cosine curve in the HR graph as you take sleep at night and show a lot of activity during the day. From cosinor analysis for the daily HR, four parameters are produced: amplitude, acrophase, mesor, and R-squared. |
|  | HR_CR_acrophase | The daily HR rhythm of cosine curve has a peak point at time t, where t is acrophase. Acrophase tells a degree of misalignment of rhythm. |  |
|  | HR_CR_mesor | Mesor is an average HR per day. The more active HR, the higher mesor. |  |
|  | HR_CR_good_of_fit | R-squared means good of fitness for the daily HR in cosine curve fitting. The more fitting well, the higher R-squared. |  |
|  | resting_heartrate | The average HR within timeslots where a user has no activity (subject’s resting). | Measuring the HR at rest is a good idea. It tends to rise when stressed, uneasy, or unhealthy, which might be correlated with mood state. |
