# Supplementary material for: Mood Prediction of Patients With Mood Disorders by Machine Learning Using Passive Digital Phenotypes Based on the Circadian Rhythm: Prospective Observational Cohort Study
Source: J Med Internet Res. 2019 Apr 17;21(4):e11029. doi: 10.2196/11029 (PMC6492069; doi:10.2196/11029)
Supplement: Multimedia Appendix 3 [file jmir_v21i4e11029_app3.docx]

**Supplementary Figure 1.** Different parameter setting affects model performance. The legend means the model test period q and the horizontal axis means the model training period p has 1 time of q days, 2 times of q days, 3 times of q days, and so on. The vertical axis means the AUC result after evaluating model performance with a parameter combination of p and q.
