# Supplementary material for: Mood Prediction of Patients With Mood Disorders by Machine Learning Using Passive Digital Phenotypes Based on the Circadian Rhythm: Prospective Observational Cohort Study
Source: J Med Internet Res. 2019 Apr 17;21(4):e11029. doi: 10.2196/11029 (PMC6492069; doi:10.2196/11029)

**Supplementary Figure 2.** The entire ROC curves of the main manuscript Figure 2 are here presented for clearer understanding of some unreported calibration in sensitivity and specificity.


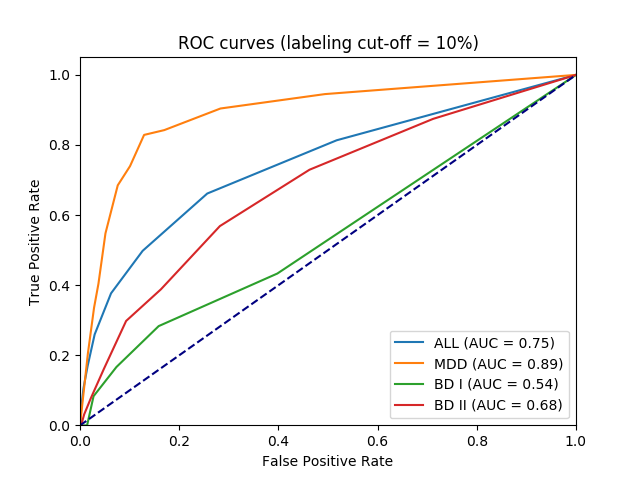


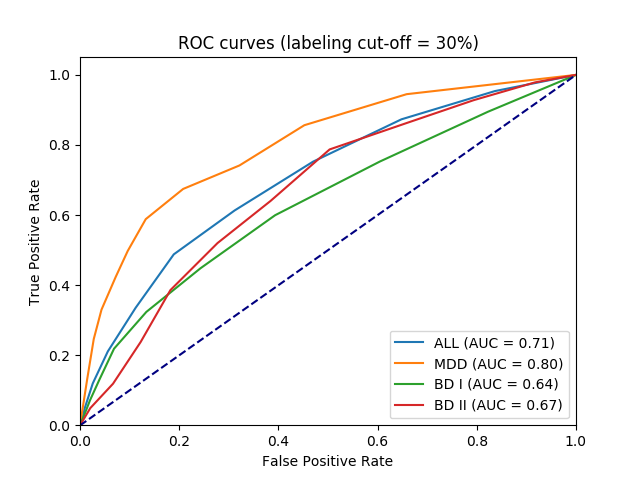

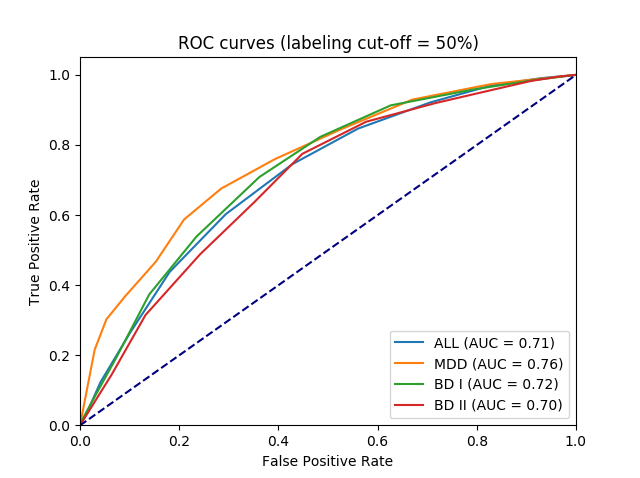

Supplement: Multimedia Appendix 4 [file jmir_v21i4e11029_app4.docx]
