# Supplementary material for: Mood Prediction of Patients With Mood Disorders by Machine Learning Using Passive Digital Phenotypes Based on the Circadian Rhythm: Prospective Observational Cohort Study
Source: J Med Internet Res. 2019 Apr 17;21(4):e11029. doi: 10.2196/11029 (PMC6492069; doi:10.2196/11029)

**Supplementary Figure 3.** This is a Kernel Density Estimate (KDE) plot describing variance of the model performance that was reported in the main manuscript Figure 2C (Mood state labeling with 50% cut-off) for the mood disorder group ALL. The horizontal axis presents the area under the curve (AUC) distribution and the vertical axis presents density of each AUC observation from multiple performance evaluation rounds. The total number of evaluation rounds is 668.


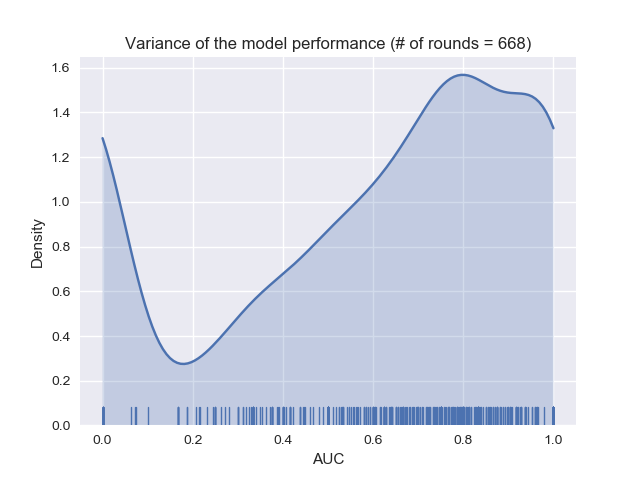

Supplement: Multimedia Appendix 5 [file jmir_v21i4e11029_app5.docx]
