# Supplementary material for: Mood Prediction of Patients With Mood Disorders by Machine Learning Using Passive Digital Phenotypes Based on the Circadian Rhythm: Prospective Observational Cohort Study
Source: J Med Internet Res. 2019 Apr 17;21(4):e11029. doi: 10.2196/11029 (PMC6492069; doi:10.2196/11029)
Supplement: Multimedia Appendix 6 [file jmir_v21i4e11029_app6.docx]

**Supplementary Table 3.** The number of samples used in the mood state and mood episode prediction.

Supplementary Table 3A - the number of samples used in the mood state prediction of Figure 2

| Group | Cut-off(%) | # of positive samples | # of total samples |
| --- | --- | --- | --- |
| ALL | 10 | 187 | 2003 |
|  | 30 | 628 |  |
|  | 50 | 1014 |  |
| MDD | 10 | 65 | 607 |
|  | 30 | 156 |  |
|  | 50 | 263 |  |
| BD I | 10 | 25 | 807 |
|  | 30 | 198 |  |
|  | 50 | 367 |  |
| BD II | 10 | 97 | 589 |
|  | 30 | 274 |  |
|  | 50 | 384 |  |

Supplementary Table 3B - the number of samples used in the mood episode prediction of Table 1

| Group | Episode | # of positive samples | # of total samples |
| --- | --- | --- | --- |
| ALL | NONE | 1852 | 2256 |
|  | DE | 278 |  |
|  | ME | 26 |  |
|  | HypoME | 100 |  |
| MDD | NONE | 683 | 738 |
|  | DE | 55 |  |
|  | ME | 0 |  |
|  | HypoME | 0 |  |
| BD I | NONE | 718 | 844 |
|  | DE | 100 |  |
|  | ME | 26 |  |
|  | HypoME | 0 |  |
| BD II | NONE | 451 | 674 |
|  | DE | 123 |  |
|  | ME | 0 |  |
|  | HypoME | 100 |  |

ALL; all kinds of mood disorders

MDD; major depressive disorder

BD; bipolar disorder

DE; depressive episode

ME; manic episode
