# Supplementary material for: Mood Prediction of Patients With Mood Disorders by Machine Learning Using Passive Digital Phenotypes Based on the Circadian Rhythm: Prospective Observational Cohort Study
Source: J Med Internet Res. 2019 Apr 17;21(4):e11029. doi: 10.2196/11029 (PMC6492069; doi:10.2196/11029)
Supplement: Multimedia Appendix 7 [file jmir_v21i4e11029_app7.docx]

**Supplementary Table 5. Prediction performance comparison between personalized model versus general model.** For example, in the case of Next three days, if the general model had the accuracy of 0.6, then the personalized model showed the accuracy of 0.838 (=0.6 + 0.238) on average.

| **Prediction scenario** | **Personalized > General** | **Personalized = General** | **Personalized < General** | **Average outperformance** |
| --- | --- | --- | --- | --- |
| Next three days | 100% | 0% | 0% | 23.8% |
| Depressive episode | 70% | 0% | 30% | 13.1% |
| Manic episode | 100% | 0% | 0% | 26.4% |
| Hypomanic episode | 100% | 0% | 0% | 24.6% |
