# Supplementary material for: Mood Prediction of Patients With Mood Disorders by Machine Learning Using Passive Digital Phenotypes Based on the Circadian Rhythm: Prospective Observational Cohort Study
Source: J Med Internet Res. 2019 Apr 17;21(4):e11029. doi: 10.2196/11029 (PMC6492069; doi:10.2196/11029)
Supplement: Multimedia Appendix 8 [file jmir_v21i4e11029_app8.docx]

**Supplementary Table 4.** The AUC performance of each partially constructed model.

The first column means a group of features that are missed in the model construction, and the second column is the number of features that are used in the model construction except the group of missing features. The rest of columns are the constructed model performance without using the missing features.

| Missing features | # of used features | Sensitivity | Specificity | Accuracy | AUC |
| --- | --- | --- | --- | --- | --- |
| None | 130 | 0.713 | 0.574 | 0.652 | 0.695 |
| Step | 110 | 0.71 | 0.579 | 0.655 | 0.684 |
| Sleep | 110 | 0.7 | 0.582 | 0.655 | 0.687 |
| Heart rate | 80 | 0.695 | 0.573 | 0.646 | 0.683 |
| Light | 110 | 0.689 | 0.569 | 0.644 | 0.683 |

AUC; area under the curve
